# Estimating seed demand in the presence of market frictions: Evidence from an auction experiment in Nigeria

**DOI:** 10.1016/j.jdeveco.2023.103242

**Published:** 2024-03

**Authors:** Tesfamicheal Wossen, David J. Spielman, Arega D. Alene, Tahirou Abdoulaye

**Affiliations:** a International Institute of Tropical Agriculture (IITA), Nairobi, Kenya; b International Food Policy Research Institute (IFPRI), Washington, DC, USA; c International Institute of Tropical Agriculture (IITA), Bamako, Mali

**Keywords:** Asymmetric information, Auctions, Seed systems, Seed certification, Cassava, Nigeria

## Abstract

This paper measures the effect of seed quality misperceptions on bidding behavior and demand for high-quality seed using an information-provision experiment within an incentive-compatible Vickery Second Price (SPA) auction mechanism that mimics seed purchasing decisions in the presence of seed market frictions. We find that most individuals are prone to quality misperception and revise their bids upwards (downwards) in response to positive (negative) quality signals. In addition, by exploiting random variation in the timing of cash grants, we show that imperfect information influences farmer seed valuation, even in the presence of potentially binding liquidity constraints. We also demonstrate that the provision of quality information does not fully resolve quality misperceptions. We then show that unresolved or persistent misperception is severe enough to distort bidding behavior, and ignoring it could lead to biased willingness-to-pay estimates. Our findings have important implications both for improving inference related to the identification and estimation of willingness to pay for quality seed in the presence of market frictions, and for the design of seed sector polices in developing countries.

## Highlights

• We investigate how asymmetric information about quality distorts farmers' valuation of seed quality.
• We use an incentive compatible auction experiment to demonstrate that most farmers are prone to quality misperceptions.
• Imperfect information about seed quality significantly reduces (increases) demand for high-quality (low-quality) seed.
• Quality misperception persists even after the provision of quality information.

## 1 Introduction

Vegetatively propagated crops (VPCs) such as cassava are central to both agricultural production systems and consumption choices throughout much of the developing world (Spielman et al., 2021; Wossen et al., 2020). These crops form an essential part of the diet of more than three billion people and provides a source of livelihood for millions of processors, and traders in developing countries (Scott, 2021; Thiele et al., 2021). In Africa, the world's largest producer of VPCs, these crops provided about 18% of the daily per capita calorie intake for more than one billion people in 2021 (FAO, 2023).^1^ In the humid tropics of Africa, where VPCs are the most important staples, the contribution of foods derived from these crops to total caloric needs ranges from about 27% in Nigeria to 69% in the Democratic Republic of Congo (i.e. the two countries account for over 50% of Africa's VPC production in 2021) (FAO, 2023; Thiele et al., 2021). But yields for these crops in Africa fall well below global averages, contributing to poverty and food insecurity (Scott, 2021). A well-established path towards yield improvement for VPCs in Africa, where productivity changes critically affect livelihoods, is increasing the availability of high-quality seed with superior genetic, physical and physiological quality traits for cultivation by small-scale, resource-poor farmers that dominate the region's agriculture sectors (Maredia and Bartle, 2022; Almekinders et al., 2019; Maredia et al., 2019). For instance, Wossen et al. (2019) find that replacing landraces with improved cassava varieties increases yield by up to 60% on average in Nigeria.

But while smallholders are often aware of the productivity benefits of high-quality seeds of improved varieties, their demand for these technologies remain surprisingly low (Bold et al., 2017, 2022; Sheahan and Barrett, 2017). This may partly reflect the low or variable returns to the technologies, or the costs in obtaining them from markets (Bold et al., 2022; Ashraf et al., 2009). Another potential explanation is the smallholder's inability to accurately assess the technology's quality due to weak market regulation and extension systems or, more generally, from market failures arising from asymmetric information (Michelson et al., 2023; Bold et al., 2022; Bai, 2021; Ashour et al., 2019; Jack, 2011). In particular, in the presence of information asymmetries about quality between sellers and farmers and given the credence nature of seed quality, neither genetic nor physical quality traits can be observed or assessed by farmers accurately at the time of exchange (Wossen et al., 2022; Michelson et al., 2020; Wineman et al., 2020). These problematic exchanges that make quality assessment difficult may allow lower-quality seed to crowd into the market, thereby crowding out demand for high-quality seed in an illustration of the classic “lemons” market (Akerlof, 1970).

Focusing on Nigeria, the world's largest producer of cassava and where imperfect information regarding seed quality is acute, this paper investigates how asymmetric information about quality distorts farmers' valuation of seed quality. Cassava is the most important crop by area and production volume in Nigeria (Wossen et al., 2020). Despite the importance of the crop and substantial investment in breeding that led to the development of more than 50 improved varieties, the seed system is almost entirely informal and unregulated, relying on farmer-to-farmer and own-saved planting material (essentially, cassava stems cut from mature plants). Wossen et al. (2017) report that while about 60% of farmers in Nigeria cultivate improved cassava varieties, almost all rely on informal farmer-to-farmer exchanges, where variety type and stem quality are often unknown or heterogenous.

In these unregulated informal markets, farmers make their seed purchasing decision based on quality proxies inferred from the physical appearance and other observable characteristics (e.g., stem color or visible symptoms of disease). However, quality beliefs inferred from partial information sets about a credence good may not be accurate, introducing a quality inference or quality misperception problem into the market which, in turn, affects individuals’ valuation of the good. Still, existing approaches to demand estimation rely on strong assumptions about complete markets, ruling out the effect of quality misperceptions due to market frictions (Berry et al., 2020; Canavari et al., 2019; Malone and Lusk, 2018; Cason and Plott, 2014). While such an approach may provide useful demand estimates, it fails to account for the cost of public investment in extension provision and regulatory enforcement required to achieve effective quality oversight and assurance (Spielman and Kennedy, 2016; Fuglie et al., 2006). This is often a concern in the design of willingness-to-pay (WTP) studies and inferences about consumer behavior that are drawn from these studies (Malone and Lusk, 2018; Cason and Plott, 2014).^2^

When quality is difficult to observe, economic theory and empirical evidence suggest that interventions such as regulations that impose quality standards can play a role in improving overall market efficiency (Maredia et al., 2019). In particular, quality assurance mechanisms can provide buyers with information on the source of the seed, specific variety, production date, expected germination rate, genetic and physical purity rates, and other indicators that signal seed quality (Spielman and Smale, 2017). There is considerable exploration of these remedies to market imperfections, particularly on the role of quality signaling through seed certification, truthful labeling, or other quality assurance mechanisms (Spielman et al., 2021; Banerji et al., 2016; Channa et al., 2019). However, such studies generally focus on seed markets for major cereals, which are quite distinct from markets for VPC seed. VPCs are typically propagated with the use of vegetative materials that result in a plant that is genetically identical to its parent. These planting materials—cuttings, buddings, tubers, or other non-seed plant parts—also tend to be highly perishable and susceptible to pests and disease, making market exchanges of quality VPC seed uniquely challenging.

In this paper, we conduct an information provision experiment within an incentive-compatible auction mechanism that mimics seed purchasing decisions made by smallholders both with and without market frictions. The information provision experiment was combined with a randomized cash grant disbursement to relax farmers' liquidity constraints. We use this experiment to address the following questions. First, how important is seed quality misperception in explaining low demand for high-quality seed? Second, to what extent and through which channels does the provision of quality information influence farmers' valuation of high-quality seed? Third, how do farmers respond to positive and negative seed quality signals, especially when their initial seed quality beliefs are wrong? Fourth, how important are liquidity constraints relative to asymmetric quality information in explaining variation in farmers' valuation of high-quality seed? We explore these questions by eliciting buyers’ actual bidding behavior using an incentive compatible two-step Vickery Second Price (SPA) auction mechanism that mimics seed purchasing decisions with and without market frictions.

We report four main findings. First, we find that imperfect information about seed quality significantly reduces (increases) demand for high-quality (low-quality) seed. Second, by comparing pre-and post-reveal bids for the same seed quality and individual, we show that participants are prone to seed quality misperceptions and tend to adjust their bids in response to the provision of quality information. In line with our prediction for bid-revision behavior in the presence of quality misperceptions, we find that actual bids increase in response to positive quality signals and decrease with negative quality signals. Third, by exploiting random variation in the timing of cash grants, we show that imperfect information influences farmer seed valuation, even in the presence of potentially binding liquidity constraints. Fourth, we demonstrate that quality misperception persists even after the provision of quality information.

This paper seeks to make three contributions to the growing literature on agricultural input markets, input quality, and the role of information. Our first contribution is to the literature on the role of information in general and quality signaling in particular (Bai, 2021; Michelson et al., 2020; Mastenbroek et al., 2021; Maredia et al., 2019; Banerji et al., 2016; Ashraf et al., 2013; Jensen, 2007). Our paper complements this strand of the literature by investigating the influence of seed quality information on the efficiency of buyers’ quality expectations and demand. We show that in the presence of asymmetric quality information, the relationship between true seed quality and the price premium that individual seed buyers are willing to pay is non-monotonic. And, in an illustration of the classic lemons problem, we show that asymmetric quality information has a demand-stimulating (depressing) effect for low-quality (high-quality) seed.

Our second contribution is to the literature on measurement error in agricultural data. Recent studies show that quality misperception can lead to biased and inconsistent estimates for a wide range of economic outcomes (Abay et al., 2023; Abay et al., 2021; Costanigro and Onozaka, 2020; Wossen et al., 2019; Malone and Lusk, 2017, 2018; Lusk and Shogren, 2007). By comparing participants' pre-and post-reveal bidding behavior for the same quality, we show that errors in individual bidding behavior is non-random, and failure to account for such inconsistencies could lead to biased and inconsistent price premium estimates. Relatedly, our study contributes to the literature on behavioral dimensions of individual decision-making processes (Michelson et al., 2020; Ashour et al., 2019; Maredia et al., 2019; Malone and Lusk, 2018; Cason and Plott, 2014). Specifically, we provide empirical evidence on individuals’ bid-revision behavior in response to both positive and negative quality signals and show how these revisions vary by the accuracy of the initial seed quality beliefs of individuals.

Our third contribution builds on existing studies (e.g., Maredia et al. (2019) and Oparinde et al. (2016)) that explore farmers’ misperceptions about either physical or genetic quality traits but have not simultaneously accounted for *both* within the same experimental settings. By disentangling the relative importance of genetic and physical seed quality attributes, we generate new evidence to strengthen the design of seed system policies in Africa for a class of crops—VPCs such as cassava, but also sweetpotato, yam, and potato, as well as bananas, plantains—that are typically overlooked in the analysis, design, and implementation of public investments and regulations. In this regard, our study contributes to an ongoing debate on seed sector development, where estimating effective demand for seeds and traits is a pertinent policy issue (Spielman and Kennedy, 2016; Fuglie et al., 2006), and to the wider discourse on technical change as a driver of agricultural productivity growth and food security (McArthur and McCord, 2017; Evenson and Gollin, 2003).

The remainder of the paper is organized as follows. Section 2 describes the research setting and the experimental design. Section 3 presents the empirical estimation strategy. Section 4 discusses the main findings and Section 5 concludes with policy recommendations and reflections for future research.

## 2 Study context and experimental design

### 2.1 Context

Nigeria is the world's largest producer of cassava, accounting for roughly 20% of the global cassava production in 2021. The crop is primarily produced by an estimated 6 million small-scale farmers as a staple food crop, and is valued for its tolerance of drought and its adaptability across the wide range of agro-climatic and soil conditions found in Nigeria (Wossen et al., 2017). It is also quickly becoming a major source of cash income for farmers who sell it as an input to agro-industrial processors preparing cassava flour (*gari*) and other products to meet growing demand from urban and rural non-farm households (Wossen et al., 2020). There are even efforts underway in Nigeria to introduce “yellow” cassava varieties that are enriched (biofortified) with Vitamin A to improve immune system health among consumers lacking other sources of the essential micronutrient in their diets (Ilona et al., 2017). As such, the crop represents a compelling entry point to enhance food security in the country.

Yet despite the importance of cassava to Nigeria's entire food system, the physiological and physical quality of cassava seed—irrespective of genetic improvement—rarely garners attention in the formulation and implementation of seed policy and regulatory reform efforts **(**Spielman et al., 2021; Wossen et al., 2020).^3^ For instance, while about 39% of the cassava area in Nigeria is under improved cassava varieties, almost all farmers rely on the informal system to obtain planting material for improved varieties (Wossen et al., 2017). Because they often transmit soil- and seed-borne pests and diseases from one generation to the next, low quality stems can significantly affect both the yield and quality of the tuberous cassava roots that are ultimately produced, consumed, and sold. The formal market for certified (i.e., quality assured) cassava seed accounts for less than 1% of the total certified seed production for all crops in the country (Wossen et al., 2020). And while genetic improvements designed to make pest- and disease-resistant cassava varieties available to farmers can mitigate some of these quality issues, they are often viewed as only partial solutions that should be accompanied by high-quality seed and better management practices. Not surprisingly, Nigeria's cassava yields are at most half of the yields achieved in other major cassava-producing countries such as Thailand and Vietnam (Spielman et al., 2021; Wossen et al., 2020).

This low productivity is partly attributable to market imperfections that constrain farmers’ access to high-quality seeds of improved cassava varieties. Cassava stems are typically exchanged in informal markets that embody many of the classic failures observed in other seed markets: asymmetric information between seller (who may know the genetic and physical potential of the seed) and farmer (who cannot assess quality prior to cultivation). For instance, while more than 50 improved cassava varieties have been officially released and disseminated to farmers in Nigeria, farmer-to-farmer exchanges are the single most important means of obtaining stems of these varieties (Wossen et al., 2017). Through these exchanges, farmers often obtain recycled stems at a fairly low price and sometimes for free (excluding transport costs), although variety type and stem quality may be unknown or may vary widely among the large number of suppliers in these informal markets.

In these informal markets, the trade in cassava stems generally precludes any form of quality assurance, apart from the transmission of information about the reputation of a particular seller who might have quality stems of existing or new cassava varieties. Moreover, due to the credence nature of stem quality, stem exchanges in informal markets are also difficult to quantify or characterize, resulting in insufficient information on the type of variety or quality of stems exchanged. Hence, farmers often acquire stems that may not be what they expect or need either because sellers have lost track of the variety type being exchanged, or because of willful deception on the seller's part (Wossen et al., 2019; Wineman et al., 2020).^4^ The absence of quality assurance contributes to low yields not only by introducing potentially pest- or disease-infected stems into the field, but by also shaping the farmer's choice of complementary inputs and management practices (Rabbi et al., 2015). The magnitude of the problem is not small: a recent review of farmer-reported and genotyped data from 16 empirical studies across 9 crops and 10 developing countries find that only 24% of farmers know the variety type they are growing (Stevenson et al., 2023). More importantly, using DNA-fingerprinting to identify improved cassava varieties in Nigeria, Wossen et al. (2022) find that 25% of farmers often misperceive improved varieties as local varieties, while 10% misperceive in the other direction. Besides direct yield losses due to low genetic, physical, and physiological seed quality, their results show that variety misperception can introduce inefficiencies in the production process by distorting the allocation of complementary inputs such as fertilizer. It is the nature and magnitude of the quality misperception issues that motivates our information provision experiment described in the next section.

### 2.2 Experimental design

The auction experiments were conducted in Benue and Oyo States of Nigeria, the heart of Nigeria's cassava production system. A total of 421 cassava farmers randomly selected from 22 villages across these two states participated in one of the 38 auction sessions that were conducted from November to December 2019, which coincides with the period when farmers make their decisions on cassava planting.

Our auction experiment was comprised of three main parts: (i) pre-auction interviews, (ii) auctions for cassava stems, and (iii) post-auction interviews. In the auctions themselves, participants bid on a bundle of cassava stems, the most common unit used to buy/sell cassava stems in the local market, for three types of cassava stem bundles in two rounds. These were (i) certified stems of an improved variety (Tropical Manihot *esculenta* (TME) 419), (ii) non-certified stems of an improved variety (recycled TME 419), and (iii) recycled stems of multiple and typically unknown local varieties (referred to in our study as “local stems”).^5^

It is important to note the following differences in the quality of the three stem types included in our auction: (i) certified TME 419 is equivalent to recycled TME 419 in terms of genetic quality, and both are genetically superior to local varieties; (ii) certified TME 419 is superior to both recycled TME 419 and the local varieties, with the latter two types being indistinguishable in terms of physical quality (according to which certified TME 419 is superior to recycled TME 419), and (iii) the combination of genetic and physical quality are consistently ordered (from best to worst) as certified TME 419, recycled TME 419, and recycled stems of local varieties. Alternatively, the quality levels of the three stem types can also be referred to as high-quality (certified TME 419), medium-quality (recycled TME 419) and low-quality (local stems).^6^ It is also worth noting that most cassava farmers in Nigeria rarely purchase certified stems from the market when they are available, but may still be aware of their prices. For references purposes, the price of certified improved stems ranged between NGN 400 and NGN 800 per bundle, while the median price of recycled (non-certified) improved stem was about NGN 250 per bundle.^7^ Hence, the price used in our auction ranges from 0 to NGN 1000 per bundle.

Our auction proceeded as follows. Upon arrival, each participant was interviewed using a structured survey instrument that covers demographic, household, and farm characteristics, as well as retrospective questions about their cassava cultivation practices and their access to and usage of stems for planting. After completing the survey, a group of participants were invited to a room in their respective village centers for the auction experiments. Instructions were read aloud prior to the main auction, and a practice auction was conducted using three soap bars of different qualities to familiarize participants with the bidding process and the auction mechanism. After the practice round, the cassava stem auctions were conducted in two rounds. We advised participants to submit their maximum bids for each stem type simultaneously, having disclosed to them that only one of the rounds and stem types would be selected randomly as the binding outcome of the auction. From this outcome, the participant with the highest bid would win the prize (the stem bundle) and makes a payment equal to the second highest bid in cash and on the spot.^8^

In the first round, we asked each participant to state their maximum bid for each stem type without revealing the true identity of the displayed stem types. That is, although participants knew that the stem types included in the auction could be certified or recycled stems as well as improved or local varieties, information about the identity of each stem type was not relayed to them. The three stem types were displayed randomly with letter labels (T1, T2, T3) during the auction without packaging to mimic observed stem-selling practices in local markets and to reduce the possibility of anchoring or ordering effects.^9^ In addition, to mimic the typical purchasing decisions that farmers make when buying stems in the local market, participants were allowed to inspect the displayed stem bundles before submitting their bids. Hence, in the first round, participants were stating their maximum bids based on their own subjective perception of stem quality. In addition, participants were also asked to state their yield expectations for each stem type given their own context, and to rank the stem types based on the physical appearance of the displayed stem bundles.^10^

After completing the first round, all participants remained in the same room and started the second round immediately. In the second round, participants were asked to state their maximum bid for each stem type based on “objective quality information” which was conveyed to them in this second round. For certified improved stems, the objective quality information was revealed through the official labeling parameters of the National Agricultural Seed Council (NASC) of Nigeria. Among others, the information relayed to participants included the identity and specific attributes of certified TME 419 stems (e.g., source of the stems, production date, variety identity, genetic and physical purity rates, resistance to disease and pest). This information was pre-coded onto information cards that were pasted on the displayed stems. For recycled TME 419, the objective quality information similarly included identity and key attributes based on data from a nationally representative cassava monitoring survey conducted in Nigeria (Wossen et al., 2017).^11^ For brevity, we refer hereafter to the pre- and post-quality information provision rounds as the “pre-reveal” and “post-reveal” rounds.

One potential concern with our experimental design is that liquidity constraints may significantly alter participants' bidding behavior (Zhang et al., 2019; Canavari et al., 2019; Davis et al., 2010).^12^ In particular, since we required participants to pay in cash on the spot if they won the auction, those facing short-term liquidity constraints could simply have submitted bids that may not reflect their true WTP. In most experimental auctions, participants are commonly endowed with unconditional cash grants (sometimes framed as a “participation fee”) to overcome such concerns (Canavari et al., 2019). However, the offer of an unconditional cash grant may be seen as a temporary reprieve from the liquidity constraint in a spot market-type exchange, and hence participants’ bid may still not accurately reflect their demand in the presence of potentially binding liquidity constraints. Moreover, the offer of an unconditional cash grant may also introduce a “house money” effect or an “experimenter demand” effect (e.g., overbidding behavior by participants driven by a sense of reciprocity or a social desirability effect). To address these concerns, we introduced variation in the disbursement timing of the unconditional cash grants by randomly assigning the 38 auction sessions into a *pre-payment* group and a post*-payment* group.^13^ Upon arrival (i.e., during the pre-auction interview), all participants received an in-kind gift (a soft drink) as a token of appreciation for their time and participation. The pre-payment groups received NGN 1000 at the beginning of the auction session and were instructed to consider this amount as their own money. The post-payment groups were not informed about the cash payments until the very end of the auction session, and had to bid using their own money; they received the NGN 1000 payment only after completing the post-auction interview.

## 3 Data and estimation strategy

### 3.1 Descriptive statistics

Table 1 reports summary statistics for our auction participants based on the pre- and post-auction interviews. Most participants in our experiment are middle-aged males with a mean household size of about 7 people and have, on average, 19 years of experience in cassava farming. They cultivate about 4 ha of farmland, of which about 50% is allocated to cassava. These figures, as well as numerous other statistics, are very similar to figures reported in a recent nationally representative cassava monitoring survey in Nigeria, suggesting some degree of external validity in this experiment (Wossen et al., 2017). While 30% of the participants were aware of certified stems in general, only 14% indicated that they had used certified stems in the last five seasons. About 48% of the participants purchased or acquired fresh cassava stems from informal sources at some point during the last five seasons. When inquired about their satisfaction with the quality of cassava stems (of any type) from their previous purchase, only 17.6% of participants stated being satisfied with its quality. Among those participants who purchased or acquired fresh cassava stems, most indicated that they did so to obtain disease-free planting material.

**Table 1:** Summary statistics.

Variables	Mean	Std. dev.	Min	Max
Age (years)	46.05	14.34	19	95
Household size	6.95	3.68	2	20
Education (years of schooling)	8.81	4.47	0	14
Gender (1 = Male, 0 = Female)	0.78	0.42	0	1
Livestock (# of cattle)	3.35	5.52	0	29
Asset value (NGN ‘000)	2.17	2.24	0.001	9.9
Access to extension (1 = Yes, 0 = No)	0.73	0.44	0	1
Access to credit (1 = Yes, 0 = No)	0.42	0.49	0	1
Aware of certified stems (1 = Yes, 0 = No)	0.30	0.46	0	1
Used certified stems (1 = Yes, 0 = No)	0.14	0.35	0	1
Sold stems in the past (1 = Yes, 0 = No)	0.13	0.33	0	1
Bought stems in the past (1 = Yes, 0 = No)	0.48	0.50	0	1
Stem cost (% from total cassava production cost)	4.68	10.67	0	75
Low stem quality belief in the market (1 = Yes, 0 = No)	0.43	0.50	0	1
Cassava area (ha)	2.62	1.30	0.37	10.64
Total cassava production (MT)	59.19	52.38	2	467
Share of cassava harvest sold (%)	70.93	22.81	0	100
Share of casava sales from total income (%)	53.37	24.26	5	100
No. of observations	421			

Moreover, when asked about their preferred rate of stem replacement conditional on the availability of certified stems, about 49% of participants stated that they would recycle their own stems for at least two seasons before replacing it with fresh stems from outside sources. When asked about why they do not replace own cassava stems more frequently, about 76% of participants indicated that they were able to maintain good-quality stems by themselves, while only 10% indicated low returns to fresh cassava stems, and only 3% indicated high stem prices as reason. Interestingly, about 43% of the participants stated that the stems sold in the local market were, in their view, low quality.

To test whether the randomization successfully balanced the above observable participant characteristics between the pre-paid and post-paid groups, we report the randomization balance test in Appendix Table A2. Reassuringly, the balancing test shows that the randomization worked as intended, with only a 5-year difference in respondent age being statistically significant (p < 0.05).

### 3.2 Empirical estimation strategy

This section describes the empirical strategy we employed to estimate the quantities of interest using our experimentally generated panel data.^14^ In our data, we observe six bids per participant. For each auction group g, participant i, stem type s, and round t, observed bids are indexed by $b_{gist}$. The index t∈{1,2} denotes pre- and post-reveal rounds, respectively.

We denote true stem quality by $q_{s}$ and s∈{1,2,3} indexes stem quality levels. Following the design of our auction experiment, we define the following three quality levels: local stems of unimproved or unknown varieties ($q_{1})$, recycled stems of the improved variety $\left(q_{2}\right)$, and certified stems of the improved variety $\left(q_{3}\right)$. With the above notations in hand, our empirical strategy is operationalized by the following regression specification:

1 $$b_{gist}=\alpha +\sum_{s=2}^{3}\beta_{s}q_{s}+{\varphi Post}_{t}+\sum_{s=2}^{3}\gamma_{s}\left({Post}_{t}*q_{s}\right)+\psi {Cash}_{g}+\eta X_{i}+\varepsilon_{gist}$$

where Post is a dummy variable indicating whether the bids were stated before the quality information round (Post=0 for t=1) or after the quality information round (Post=1 for t=2). $X_{i}$ captures the socio-economic characteristics of the participants and their households and farms. The variable Cash is an indicator of the timing of the randomized cash payments and is coded as 1 for the pre-paid group and 0 for the post-paid group. $\varepsilon_{gist}$ is an idiosyncratic error term.

Our parameters of interest are $\beta_{s}$, φ, and $\gamma_{s}$. The parameters $\beta_{s}$ and $\left(\beta_{s}+\gamma_{s}\right)$ measure the price premium that participants are willing to pay for certified and recycled improved stems relative to the local stems before and after the provision of quality information, respectively. We use local stems as the base category to directly quantify the price premium that participants are willing to pay for certified and recycled improved stems relative to local stems. Our specification is estimated with and without the inclusion of participant-level fixed effects ($\tau_{gi}$).^15^

We also examine the extent of the adverse selection problem (i.e., quality misperception) along *physical* and *physiological* quality traits (i.e., between $q_{3}$ and $q_{2}$), genetic quality traits (i.e., between $q_{2}$ and $q_{1}$), and both genetic and *physical*/*physiological* quality traits (i.e., between $q_{3}$ and $q_{1}$). The key test for the existence of adverse selection in our data is the sign and significance of $\beta_{s}$ across the three stem types. For instance, in the presence of misperception along *physical* and *physiological* quality traits only, the difference in participants’ pre-reveal average bids between $q_{3}$ and $q_{2}$ are expected to be statistically insignificant, but the difference in their pre-reveal average bids between $q_{2}$ and $q_{1}$ are expected to differ significantly (i.e., $\beta_{31}\equiv \beta_{21}\text{;}$ and $\beta_{21}>\beta_{11}$). On the other hand, in the presence of *both* genetic and *physical*/*physiological* quality misperceptions, the difference in participants’ pre-reveal average bids between the three stem types are expected to be statistically insignificant (i.e., $\beta_{31}\equiv \beta_{21}\text{;}$ $\beta_{31}\equiv \beta_{11}$ and $\beta_{21}\equiv \beta_{11}$).^16^

In what follows, we examine an important mechanism through which the provision of quality information is expected to influence the price premium that individuals are willing to pay for a given stem quality: the quality misperception mechanism. In this mechanism, participants are expected to revise their bids in response to their own quality prediction error (i.e., when participants are prone to quality misperceptions). To do so, we introduce, $p_{is1}$,which denotes participant i’s pre-reveal quality ranking of stem type s (from worst to best) based on the rank order of their pre-reveal bids. Following the same quality ordering logic of $q_{s}$, participants' pre-reveal bids were discretized to take three values to ensure that their highest and lowest bids match with their best ($p_{i31}$) and worst ($p_{i11}$) stem quality perceptions, respectively.^17^ We then employ the following reduced-form parametric regression to estimate the extent of bid revision operating through a quality misperception mechanism:

2 $$\Delta b_{gis}=\xi +\mu_{s}\left(q_{s}-p_{is1}\right)+\epsilon_{gis}^{fd}$$

Eq. (2) relates the extent of participants’ bid revision with their own stem-quality prediction error and hence provides a formal test for bid revision through a quality misperception mechanism. For each stem type and participant, ${\Delta b}_{gis}$ (the difference between post-and pre-reveal bids) measures the extent of bid revision (${\Delta b}_{gis}=b_{gis2}-b_{gis1}$). As mentioned above, the difference between the true and perceived stem quality rank $(q_{s}-p_{is1}$) serves as a measure of stem quality prediction error. This variable assumes a value of zero when there is no quality misperception and positive (negative) value for under- (over-) estimation of true stem quality. Our parameter of interest, $\mu_{s}$, measures the extent of bid revision operating through a quality misperception mechanism (i.e., revision in response to own quality prediction error). Alternatively, one can interpret $\mu_{s}$ as a measure of the cost that participants are willing to incur to overcome stem quality misperception (i.e., asymmetric stem quality information). When farmers' valuations of stems are distorted by quality misperceptions, we expect a strong correlation between their bid revision and the sign of their quality prediction error. Moreover, the sign of farmers' bid revision is expected to match with the sign of their quality prediction error: upward (downward) bid revision for under- (over-) estimation of certified (recycled) stems in our setting.^18^

Finally, we supplement the above mean comparisons with non-parametric distributional equality tests, for instance, to accommodate the possibility that in the presence of mean-preserving bid dispersion across rounds, the pre-and post-reveal bid distributions could differ even if the means are identical. We implement our non-parametric distributional equality tests in three ways. First, for each round, we test equality of the cumulative distribution function (CDF) of bids between stem types as well as payment treatment groups (i.e., between group comparisons). Second, within each stem type and across all stem types, we test the equality of the pre-and post-reveal CDF of bids (i.e., paired/matched comparisons). Third, we test the equality of the CDF of bids across stem type, payment treatment, and auction round combinations simultaneously.

## 4 Results

### 4.1 Information provision and average WTP for quality stems

**Fig. 1:**
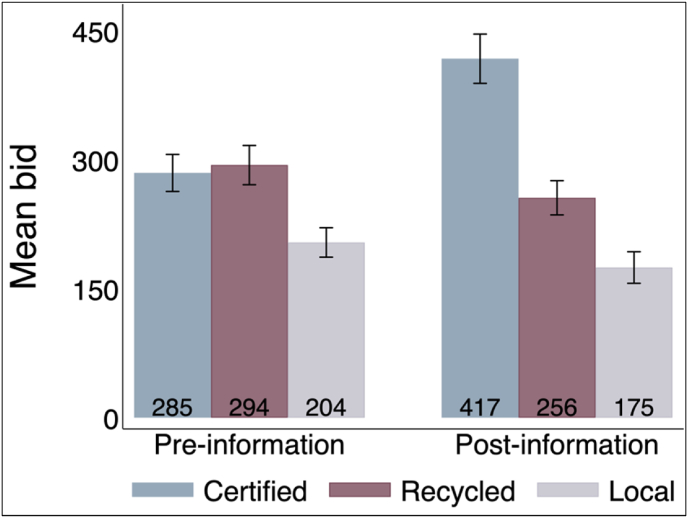
Unconditional average bid by stem type.

In this section, we describe participants' pre-and post-reveal bidding behavior. For each stem type, Fig. 1 shows the pre-and post-reveal average bids. First, we see that the pre-reveal average bid for certified and recycled improved stems track closely, indicating that farmers may not be able to distinguish the two. In particular, prior to the provision of quality information, participants’ average bid for certified and recycled improved stems was about NGN 285/bundle and NGN 294/bundle, respectively. The difference between these means is statistically insignificant at any reasonable significance level.

However, participants' pre-reveal average bid for local stems is significantly lower compared to both certified and recycled improved stems, suggesting that at least they can recognize differences between local and improved varieties, but not between certified and recycled improved stems. Specifically, participants’ average pre-reveal bid for local stems was about NGN 80/bundle and NGN 90/bundle lower relative to their average pre-reveal bid for certified and recycled improved stems, respectively. The difference in the average pre-reveal bid difference of certified and recycled improved stems vs. recycled improved and local stems is statistically significant. This result suggests that the potential for adverse selection is much higher along *physical* and *physiological* quality characteristics (i.e., between certified and recycled improved TME 419 stems) than genetic quality characteristic (i.e., between recycled improved TME 419 stems and local variety stems).^19^

Second, post-reveal, the average bid for certified improved stems increased by 46% to NGN 417/bundle (from NGN 285/bundle pre-reveal), while the average bid for recycled improved stems declined by 13% to NGN 256/bundle (from NGN 294/bundle pre-reveal). It is also worth noting that the average pre- and post-reveal bids for recycled improved stems are, respectively, 17% and 1% higher than the current market price of recycled improved stems at NGN 250/bundle. This result provides further support for the external validity of our results as participants' average bid for recycled improved stems is consistent with the current market price observed in actual informal stem markets in Nigeria. (And, as discussed in the previous section, most of our auction participants have some level of market experience with recycled improved stems). Meanwhile, the same pre- and post-reveal bids for certified improved stems are, respectively, 43% and 16% lower than the current market price of certified stems at NGN 500/bundle. Without the possibility of being deceived, one would expect participants' bids for certified improved stems to be, on average, higher than the market price. Thus, the low bids suggest either that the farmers did not trust the information or that they had already low demand for certified improved cassava stems. To provide a broader perspective and some empirical support to this mechanism, we compare participants' average post-reveal bid for certified improved stems with their highest bid (i.e., based on the highest bids of each participant across the two rounds, irrespective of the true stem type). We find that, participants’ bid for their best-valued stem is, on average, higher than the market price of certified improved stems. This result suggests that some farmers did not trust the stem quality information provided to them. We discuss this further in Section 4.4.

**Fig. 2:**
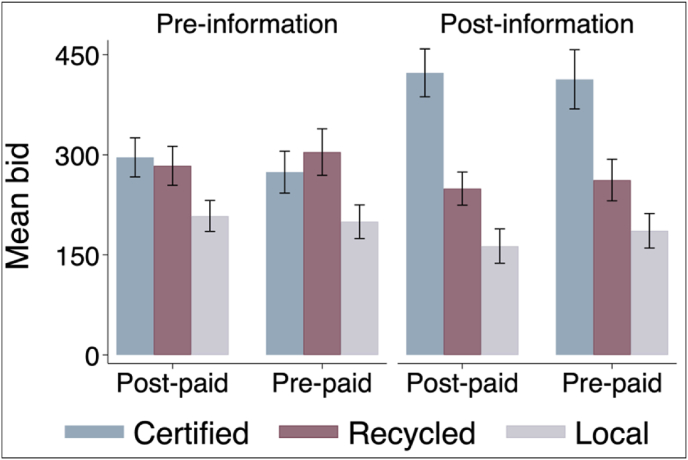
Unconditional average bid by payment treatment status.

Following the same logic, we report the basic effects of the cash payment treatment in Fig. 2. Comparing average bids across the two groups, we observe no significant difference between the groups that received the cash grant at the beginning and end of the auction session (p = 0.79 for pre-information; p = 0.35 for post-information).^20^

### 4.2 Regression results

The results reported in the previous section indicate that the provision of information about stem quality may shift participants’ WTP for high-quality stems. Our aim in this section is to confirm these initial findings while accounting for confounding factors, both observable and unobservable, to the extent possible. Column (1)–(2) of Table 2 report ordinary least squares (OLS) estimates with and without participant fixed effects. Column (3)–(6) report estimates from specifications that consider the censoring of bids at zero (i.e., either as a true zero or a censored zero). In our sample, about 8.8% of the participants stated a zero bid (6.9% pre-reveal and 10.5% post-reveal). Thus, Column (3) reports estimates excluding data points from eight participants (a total of 48 observations, or 1.9% of the full sample) who submitted a zero bid for all stem types in both the pre-and post-reveal rounds. These participants are assumed to be “true zero type” individuals who would always bid zero irrespective of information about quality. In column (4), we report estimates from a two-part or double hurdle model, which permits the zero bids to be considered as true zeros instead of censored zeros. Columns (5)–(6) report Tobit model estimations.

**Table 2:** Estimates of mean WTP for cassava stems.

Dependent variable: absolute bid value (NGN/bundle)	1	2	3	4	5	6
Certified stems	80.93***	80.93***	82.50***	75.93***	87.80***	89.99***
	(12.26)	(12.21)	(12.37)	(12.19)	(12.83)	(13.49)
Recycled stems	90.10***	90.10***	91.85***	82.73***	97.72***	101.17***
	(12.27)	(12.22)	(12.58)	(11.95)	(13.12)	(14.03)
Post-reveal (1 = yes)	−29.10***	−29.10***	−29.66***	−10.62	−42.42***	−43.30***
	(9.65)	(9.61)	(9.79)	(10.99)	(10.98)	(11.37)
Certified post-reveal	162.04***	162.04***	165.18***	148.18***	176.86***	186.30***
	(16.68)	(16.61)	(17.26)	(16.81)	(18.09)	(19.76)
Recycled post-reveal	−9.02	−9.02	−9.19	−14.52	−1.27	−3.37
	(10.34)	(10.30)	(10.51)	(10.49)	(11.44)	(12.15)
Constant	97.64*	203.91***	207.86***	114.09**	77.94	74.16
	(54.39)	(9.12)	(9.35)	(56.08)	(59.52)	(61.41)
Base category	Local	Local	Local	Local	Local	Local
Participant characteristics	Yes	No	No	Yes	Yes	Yes
Payment status	Yes	No	No	Yes	Yes	Yes
Participant FE	No	Yes	Yes	No	No	No
N	2526	2526	2478	2526	2526	2526
**Between-stem type bid equality tests**						
**Pre:**E[Certified−Recycled]	−9.17	−9.17	−9.35	−6.80	−9.92	−11.18
	(11.63)	(11.59)	(11.83)	(11.30)	(12.13)	(12.81)
**Post:**E[Certified−Recycled]	171.06***	171.06***	174.37***	162.69***	178.13***	189.68***
	(18.62)	(18.54)	(19.30)	(18.52)	(19.76)	(21.80)
**Within-stem type comparison: Single difference estimates**						
Certified (Post-Pre)	132.95***	132.95***	135.52***	137.18***	134.54***	145.71***
	(15.43)	(15.24)	(15.55)	(15.79)	(15.78)	(17.88)
Recycled (Post-Pre)	−38.11***	−38.11***	−38.85***	−25.45**	−43.66***	−46.47***
	(10.95)	(10.82)	(10.51)	(11.22)	(11.83)	(12.55)

*Notes:* Standard errors clustered at the auction session level in parentheses; *p < 0.10, **p < 0.05, ***p < 0.01. The dependent variable in all columns is the absolute bid value. In Column (3), eight observations with zero bid values for all stem types in both the pre-and post-reveal rounds are dropped. In Column (4), two-part or double hurdle model is estimated, which permits the zero and non-zero mixture types to be generated by different selection/decision processes. In column (5)–(6), estimates are reported from a Tobit model that accounts for censoring at 0 and at 0 and 1000, respectively.

Estimates reported in Table 2 suggest that pre-reveal, participants’ mean bid for certified and recycled improved stems is, respectively, about NGN 81/bundle and NGN 90/bundle higher than their mean bid for local stems. This translates into mean bids that are, respectively, 54% and 58% higher than the mean bid for local stems, all else being equal. Following the provision of information about stem quality, participants increased their mean bid for certified improved stems by about NGN 161/bundle relative to local stems. This indicates that the price premium that participants are willing to pay for certified improved stems compared to local stems increased from a pre-reveal NGN 81/bundle to NGN 242/bundle post-reveal.

Moreover, the between-stem type bid equality test for certified relative to recycled improved stems reported at the bottom of Table 2 shows a negative but statistically insignificant difference prior to the provision of quality information but highly positive and significant following the provision of stem quality information. This result suggests that with the provision of quality information, participants are willing to pay considerably more for certified improved stems relative to both recycled improved stems and recycled local stems. We note that the difference in the estimated price premia with and without the provision of quality information is economically meaningful: the price premium that participants are willing to pay for certified improved stems relative to recycled improved stems increased from zero to about 170 NGN/bundle, which represents about 34% of the market price for certified improved stems at 500 NGN/bundle.

Relatedly, the within-stem type single-difference estimates show that participants are willing to pay considerably more for high-quality stems post-reveal relative to pre-reveal as participants increase their bids for certified improved stems significantly, while at the same time reducing their bids for recycled improved stems and local stems. Together, our results suggest that demand for certified improved stems will be low in the presence of market frictions (i.e., asymmetric information about stem quality) and that the provision of quality information will have a strong demand-stimulating effect for high-quality (i.e., certified) stems. Conversely, quality misperceptions due to asymmetric quality information may increase demand for lower-quality stems by depressing demand for higher-quality stems.^21^

**Fig. 3:**
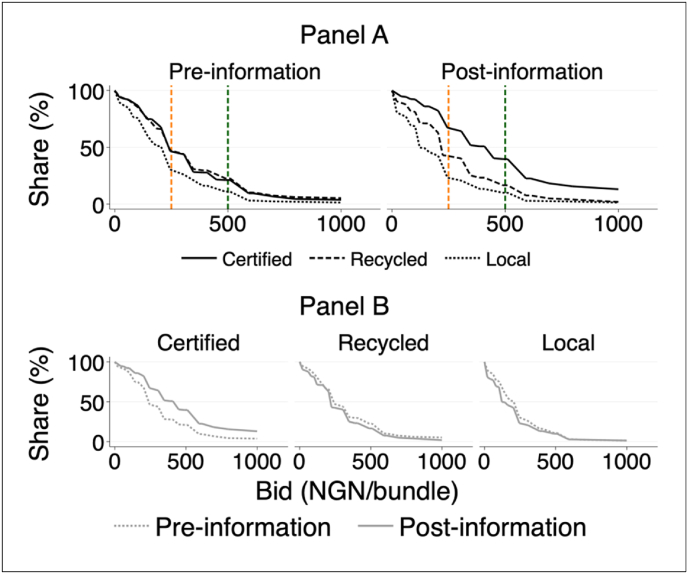
Round- and stem-specific demand curves. *Notes:* For each stem type, Panel A shows the distribution of participants pre-reveal bids (top left graph) and post-reveal bids (top right graph). For each stem type, Panel B displays the matched pre-and post-reveal bid dsitributions. Pre-information: the Kolmogorov-Smirnov (KS) D statistic on the test of equality of the demand curves of (a) certified vs. recycled improved stems is 0.026 (p = 0.999); (b) certified improved stems vs. local stems is 0.178 (p = 0.0001); (c) recycled improved stems vs. local stems is 0.181 (p-value = 0.0001) and (d) Kruskal-Wallis chi-squared statistic on the test of quality of the three demand curves simultaneously is 46.7 (p = 0.0001). Post-information: (a) certified vs. recycled improved stems(p = 0.000); (b) certified improved stems vs. local stems (p = 0.000); (c) recycled improved stems vs. local (p-value = 0.000) and (d) Kruskal-Wallis chi-squared statistic on the test of quality of the three demand curves simultaneously is 189.5 (p = 0.0001). P-values on the test of equality of the within stem type pre-and post-reveal bid distributions: (a) certified (pre-vs. post-reveal: p = 0.000); (b) recycled (pre-vs. post-reveal: p = 0.004); and (d) local (pre-vs. post-reveal; p = 0.000).

Next, we formally test the equality of the distribution of the seed-quality specific pre-and post-reveal bids. In doing so, we examine whether the pre-and post-reveal bid distributions yield statistically identical demand curves. That is, instead of mean comparisons (as reported in Table 2), we compare the entire cumulative distribution function (CDF) of bids while controlling the probability of any false positive or familywise error rate (FWER) following the approach of Goldman and Kaplan (2018) and Kaplan (2019). In this case, the null hypothesis is that the two CDFs are identical at all points, which may not be true even if the conditional means are identical. Panels A and B of Fig. 3 illustrate the between- and within-stem type inverse demand curves generated from the bids of all 421 participants, respectively. The left and right panels of Fig. 3 (Panel A) display the pre- and post-reveal demand curves, respectively. For each round, stem type and auction price point (p) are reported along the x-axis, and the share of participants whose bid was equal or greater than p is reported on the y-axis. Pre-reveal (the top left graph in Panel A), most participants were able to distinguish between improved and local stems but not between certified and recycled improved stems.

The Kolmogorov-Smirnov (KS) test rejects equality of the certified improved stem and local stem demand curves (p = 0.0001) as well as recycled improved stem and local stem demand curves (p = 0.0001). However, the demand curves of certified and recycled improved stems lie effectively on top of each other, and the KS test fails to reject equality of the two demand curves (p-value = 0.999). This indicates that at the same price, while demand for local stems was low, demand for certified and recycled improved stems was almost identical at any price point. In fact, at the current market price of certified improved stems (500 NGN/bundle), about 21% and 22% of the participants were willing to pay a positive premium for certified and recycled improved stems, respectively. Fig. 3 (the top right graph in Panel A) shows that the provision of quality information shifts the full distribution of participants’ bids for certified improved stems in the intended direction, and the demand curve for certified improved stems almost always achieves first-order stochastic dominance over recycled improved stems. More importantly, we also reject equality of the post-reveal demand curves between certified and recycled improved stems as well as between recycled improved stems and local stems at all reasonable significance levels (p < 0.0001).^22^ Post-reveal, the share of participants who are willing to pay a premium for certified improved stems increased from 21% pre-information to about 40% post-information.

For each stem type, Panel B of Fig. 3 displays demand curves constructed from the matched pre-and post-reveal bids of the same individuals (i.e., for the same seed type and individual across the two auction rounds). For certified improved stems, the demand curve clearly shifts to the right following the provision of quality information. In contrast, the demand curve for recycled improved stems and local stems shifts to the left following the provision of quality information. For all stem types, we reject the null of equal distributions.^23^ Following the same logic, Figure A2 in the appendix shows the round-specific bid distributions by payment treatment status. Pre-reveal, we reject the null of identical distributions between the two groups. However, we fail to reject the null of identical distributions between the two groups post-reveal. As expected, the bid revision distributions remain essentially the same between the pre-paid and post-paid group and we fail to reject the null of equal distributions.^24^

Finally, since the distribution of participants' pre-and post-reveal bids provides information about the likely stem demand that would emerge in the market with and without a credible certification system, we also use the experimental results to rationalize the patterns we observe in the actual informal stem markets in Nigeria. Taking the post-reveal bids as proxies for farmers' true valuations of the three stem types, we see that local stems are about correctly valued, as are improved non-certified stems (medium quality). However, improved certified stems (high quality) are massively under-valued. This suggests that under-provision of high-quality stems would be ubiquitous in largely unregulated and informal stem markets, where farmers’ ability to infer quality is severely limited and hence stem quality is unobservable at the point of exchange. This finding provides an explanation for the lack of high-quality stem provision observed in actual informal stem markets in Nigeria. As discussed in Section 2, almost all cassava stems are traded through the informal system, with an infinitesimally small share of the overall stem exchanges moving through the formal seed system.^25^ More broadly, in terms of adverse selection and in the absence of any additional form of credible quality certification or any other quality signalling mechanisms, the above results suggest that there should be two markets with distinct prices: one for local variety stems (low quality) and one for recycled improved variety stems (medium quality), while the market for certified stems of improved varieties should shut down (i.e., unless certification is costless).

### 4.3 Evidence of systematic quality misperception and bid revision

**Fig. 4:**
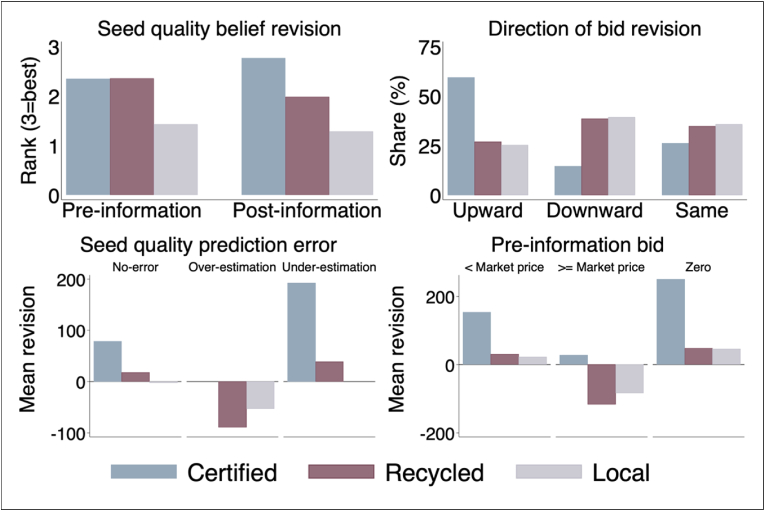
Systematic quality misperception and directional shifts in bid revision.

In the previous section, we have shown that for a given seed quality, individuals' pre-and post-reveal bids differ significantly, suggesting the presence of systematic quality misperceptions and bid-revision behavior in our data. To demonstrate this claim, we report the directional shift of participants' stem quality-specific bids in Fig. 4. Fig. 4 reveals three important aspects of participants’ bidding behavior. First, stem quality misperception (i.e., quality prediction error), measured as the difference between the true stem quality indicator ($q_{s}$) and the submitted bid rank orders ($p_{is1}$), is substantial in our data. This is indicated in the top left panel of Fig. 4.

The Spearman rank correlation, applied here as a nonparametric measure of the monotonic ordinal associations between $p_{is1}$ and $q_{s}$, increased from 0.41 pre-reveal to 0.74 post-reveal. This suggests that farmers value quality stems and their valuations are distorted by quality misperception. We also note that the Spearman rank correlation coefficient remains less than 1 post-reveal, suggesting that quality misperceptions persist even after the provision of information about stem quality, as will be discussed further in Section 4.4. Note that, if the provision of quality information fully resolves quality uncertainty (misperception), the rank order of post-reveal bids should be identical with the true stem quality signal conveyed to them (i.e., the average rank order of post-reveal bids is expected to be 3 for certified improved stems).^26^ Second, post-reveal, more than 85% of the participants’ post-quality information bids for certified improved stems were equal to or higher than their pre-quality information bid, suggesting a positive correlation between stem quality and bid revision (the top left panel of Fig. 4). Third, participants were more likely to revise their bid upwards (downwards) when under- (over-) estimating true stem quality (bottom left panel of Fig. 4). Moreover, for all stem types, participants were also more likely to revise their bids upwards when their pre-reveal bids are below the market price (bottom right panel of Fig. 4).

**Fig. 5:**
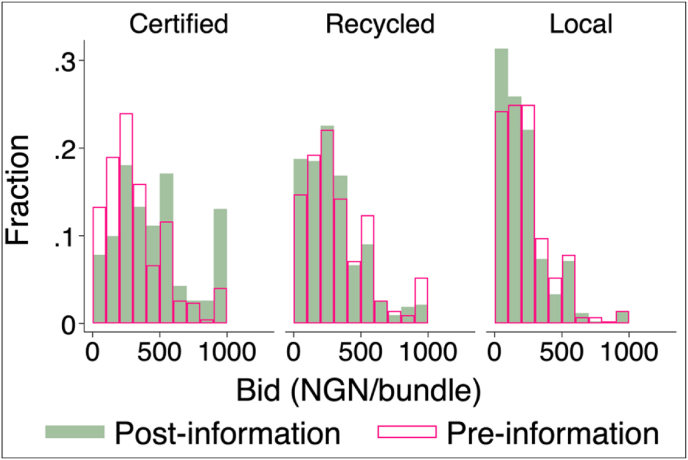
Distribution of pre-and post-information bids.

To supplement the above mean comparisons, we characterize the full distribution of participants’ quality-specific pre- and post-reveal bids (i.e., from NGN 0 to 1000/bundle).^27^ As illustrated in Fig. 5, when presented with quality information, participants revise their bids in response to the quality signal conveyed to them along the entire bid distribution. This can be illustrated by the within-stem type comparison in Fig. 5, which suggests that individuals respond to the quality information signal in a way that is consistent with the presence of quality misperceptions in the sense that their post-reveal bidding patterns are highly correlated with the true stem quality signal conveyed to them. Note that pre-reveal, participants were bidding based on their own subjective stem quality perception as information about the true quality of each stem type was not conveyed to them. If individuals were not prone to quality misperception, the systematic bid revision behavior reported in Fig. 5 would not be observed in our data.

Table 3 shows the regression equivalent of the graphical arguments presented in Fig. 4, Fig. 5. Following our empirical strategy, we regress bid revisions on quality prediction error to quantify the information-induced absolute bid revision rates through a quality misperception mechanism.^28^ We also consider a regression of the log of bid revision on quality prediction error to estimate the magnitude of the relative bid revision rate. In addition, to avoid bias due to an “averaging-out” effect across stem types, we decompose quality prediction error into over-estimation and under-estimation so that a positive (negative) error indicates that the participant under-estimates (over-estimates) stem quality. We find that under-estimation of stem quality (relative to no quality prediction error) leads to up to 73% upward bid revision, while over-estimation results in up to 65% downward bid revision (relative to no quality prediction error).^29^

**Table 3:** Estimates of bid revisions conditional on own quality prediction error for cassava stems.

Dependent variable: Bid revision value	Absolute bid revision			Relative bid revision		
	1	2	3	4	5	6
Over-estimation $(q_{s}-p_{is1}<0$)	−125.61***	−127.59***	−132.44***	−0.58***	−0.63***	−0.65***
	(14.40)	(17.44)	(18.95)	(0.14)	(0.15)	(0.16)
Under-estimation ${\begin{array}{c} (q \end{array}}_{s}-p_{is1}>0$)	123.07***	138.50***	135.15***	0.61***	0.73***	0.71***
	(15.33)	(21.32)	(21.73)	(0.12)	(0.17)	(0.17)
Base category	No prediction error:					
Participant FE	No	Yes	Yes	No	Yes	Yes
N	1263	1263	1239	1263	1263	1239

*Notes:* Standard errors clustered at auction session level in parentheses; *p < 0.10, **p < 0.05, ***p < 0.01. Absolute bid revision is computed as $\Delta b_{gis}=b_{gis2}-b_{gis1}$ and relative bid revision as $\Delta \text{ihs}b_{gis}={ihsb}_{gis2}-{ihsb}_{gis1}$. Relative changes are calculated using an inverse hyperbolic sine (IHS) transformation. We favored IHS over logarithmic transformation as some participant's bid is zero, the log of which is not defined. Note that quality under-estimation is only possible for certified and recycled improved stems but not for local stems since it is the lowest possible quality in our experiment. Similarly, over-estimation is only possible for local and recycled stems since certified stem is the highest possible quality in our experiment. In Column (3) and (6), observations with zero bid values are dropped for all stem types in both the pre-and post-reveal rounds. In all columns, a rank tie dummy is included to account for ties or duplicates in the rank order of participants bid ($p_{is1})$.

### 4.4 Persistence of stem quality misperception

**Fig. 6:**
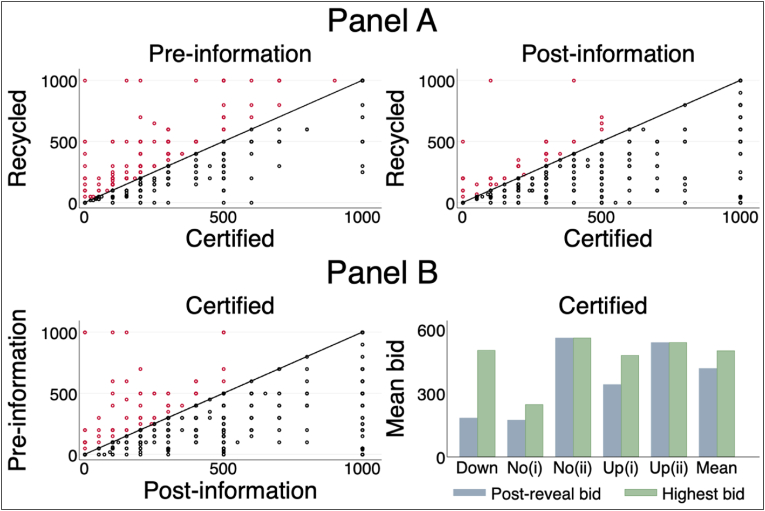
Persistence of stem quality misperception.

Although we have shown that participants revise their bid upwards (downwards) for high-quality (low-quality) stems in response to information provision, not all participants do so. To demonstrate this further, we report the distribution of “compliers” and “defiers” in our data by tracking the stem quality-specific bidding behavior of the same individual following the approach of de Quidt et al. (2018). In our context, compliers are those individuals that logically updated their bids, where logical bid revision is defined as revising upwards when under-estimating certified improved stems. For certified improved seed, strict compliers are expected to lie below the 45-degree line in Fig. 6 (i.e., in the top right and bottom left graphs). On the other hand, defiers (i.e., those who behaved opposite to the quality-information signal conveyed to them) are expected to lie above the 45-degree line. Our comparisons in Fig. 6 involve the following: First, for each round, we compare the certified and recycled improved stem bids of the same individual in Panel A.^30^ Second, focusing on certified improved seed, we compare the pre-and post-reveal bids of the same individual in Panel B (i.e., the bottom left graph of Fig. 6). Finally, we compare the average post-reveal bids of compliers and defiers (bottom right graph in Panel B of Fig. 6).

Two key results are worth highlighting. First, Fig. 6 shows that (a) post-quality information, most participants' bid for certified improved stem is higher or equal to their bid for recycled improved stem (the black dots in Panel A) and (b) for certified improved seed, almost all participants’ revised their bids logically (the black dots in the bottom left graph of Panel B). Second, a considerable share of participants ignored the stem quality information conveyed to them. For certified improved stems, bid revision rates were as follows: upward revision (59.4%), downward revision (14.5%),^31^ and no revision (26.1%). Of the 110 participants (i.e., 26.1% of the total sample) who did not revise their bids for certified improved stem, 41% (i.e., 45 individuals) did not under-estimate certified improved stems initially and hence were not prone to quality misperception (i.e., No(ii) group in the bottom right graph of Panel B). For this group, their average post-reveal bid was NGN 560/bundle. The remaining 65 participants ignored the quality information conveyed to them and continued to under-estimate certified improved stem (i.e., No(i) group in Fig. 6).

Interestingly, most defiers (i.e., the 61 individuals that revised their certified improved stem bids downwards) are willing to pay considerably more for their best-valued stem (i.e., based on their highest bid, irrespective of the true stem type). These seemingly illogical revisions were also uncorrelated with participant characteristics. Still, such downward bid revisions and non-responsive behavior beg further discussion. Unfortunately, our experiment was not designed to empirically disentangle the specific behaviors that might drive these observed anomalies. Rather, our aim was to test whether stem valuations changes based on unobserved (to the farmer) stem quality, and we do not claim that information is the only variable that can affect stem valuations from one round to the next.

Regardless of the causes of the existence and persistence of misperceptions, failure to account for misperceptions could lead to biased and inconsistent price premium estimates. To this end, we examine whether the remaining quality misperception is severe enough to distort the price premia estimates reported earlier in Table 2 using non-parametric hypothesis testing approaches (Lee and Wilhelm, 2020; Wossen et al., 2022). Non-parametric test results, based on the Cramer-von Mises test statistics (*CvM*) and Kolmogorov-Smirnov test statistics (*KS*), consistently reject the null of no misperception post-reveal at all reasonable significance levels (see Appendix Table A6). These test results imply that inference via $q_{s}$ (as reported in Table 2) will lead to price premium estimates that could be biased in a meaningful way. To corroborate this claim, we report the average post-reveal bids of strict compliers (i.e., those who revised their bids for certified improved stems logically, certified improved stem being their best valued stem). As shown in Fig. 6 (i.e., Up(ii) group in the bottom right graph of Panel B), the average post-reveal bid of NGN 538/bundle among strict compliers is 8% higher than the market price of certified improved stems and 29% higher relative to the average bid of NGN 417/bundle for certified improved stem.

## 5 Conclusions

In this paper, we empirically examine how market frictions, particularly those related to asymmetric information about product quality, influences demand for certified planting material for cassava, a critically important food staple in Nigeria. We use an incentive compatible auction experiment to demonstrate that most smallholder farmers in our sample are prone to quality misperceptions and tend to revise their WTP bids upwards (downwards) in response to positive (negative) quality signals, implying that the provision of information about quality can improve demand for certified improved stems. By exploiting random variation in cash grants, we show that imperfect information influences farmers’ valuations of both high and low-quality cassava stems, even in the presence of potentially binding liquidity constraints. We also demonstrate that the provision of quality information does not fully resolve quality misperceptions.

From a policy perspective, we provide evidence on whether the reduction of information asymmetries in input markets improves overall demand for quality seed of improved varieties. Our findings have potential implications beyond cassava stems to planting materials for other VPCs for which markets have been slow to emerge in Africa south of the Sahara, partly due to the unobservable quality issues discussed at the outset of this paper. With our experimental evidence showing that farmers are prone to quality misperception and that the provision of quality information significantly shifts demand for certified improved seed, we suggest the need for greater investment in the design of quality assurance systems for VPCs that deliver seed which is simultaneously superior in genetic, physical, and physiological characteristics. This is a potentially important entry point for efforts to increase VPC productivity—yields, output, and returns—among smallholders. To date, most policy-relevant research conducted in this area has focused on farmer responses to improvements in either genetic quality or physical/physiological quality without distinguishing between them. This is partly due to the focus of research on cereal varieties and seeds (e.g., Tjernström et al., 2021; Wineman et al., 2020; Bold et al., 2017), where genetic quality issues are often more salient than physical and physiological qualities, such as soil- and seed-borne pests and diseases that are common challenges for VPC seed.

The success of such quality assurance systems depends on their capacity to encourage both varietal turnover and quality seed use by smallholders. This poses a significant challenge to policymakers and regulators who must balance their efforts to bar the entry of poor-quality seed producers against efforts to encourage new market entrants and growth in market value. The success of high-quality sellers may, in turn, depend on the response of farmers to suppliers’ investment in specializing in high-quality seed, establishing credible reputations, and capitalizing on the positive signal provided by regulatory approvals. However, current approaches to VPC seed regulation in Nigeria and many other countries in Africa south of the Sahara seem to focus more on strictly regulating the market and preventing the entrance of low-quality sellers, rather than on creating quality assurance systems that attract suppliers (Spielman et al., 2021; Wossen et al., 2020).^32^

In terms of future research priorities, this study can be extended in several ways. First, even when market frictions are addressed, adoption of higher-quality seed and improved varieties of VPCs might not occur due to considerable variation in productivity, profitability, or other factors that shape farmers' choices, including behavioral factors. In this regard, rigorous measurement of the productivity gains associated with the use of quality seed and/or improved varieties under farmers' real-world condition will be key to examining the commercial viability of alternative quality assurance mechanisms. Second, although our empirical strategy exploits within-participant variation viz the accuracy of beliefs, it would be useful to extend our auction design by randomly allocating participants into different seed quality information and quality type treatments. Third, exploring behavioral and non-behavioral mechanisms that may help explain the existence and persistence of misperceptions’ is a promising area of future research.

Finally, it would be important to expand this line of research to study supplier behavior in greater depth, especially with respect to incentive mechanisms that might cause them to invest in developing their reputation as sellers of high-quality seed. This is a growing area of research that shifts the conversation from supply-driven interventions such as seed production, regulation, and distribution, to demand-driven interventions such as improved marketing strategies for inputs (e.g., Miehe et al., 2023) and generating demand for quality inputs through more salient signals from consumers and processors (Magnan et al., 2021; Hoffmann et al., 2020).

## CRediT authorship contribution statement

**Tesfamicheal Wossen:** Conceptualization, Data curation, Formal analysis, Investigation, Methodology, Software, Visualization, Writing - original draft, Writing - review & editing. **David J. Spielman:** Conceptualization, Funding acquisition, Supervision, Writing - original draft, Writing - review & editing. **Arega D. Alene:** Funding acquisition, Supervision, Writing - review & editing. **Tahirou Abdoulaye:** Funding acquisition, Supervision, Writing - review & editing.

## Data Availability

Data will be made available on request.

## Footnotes

1 VPCs include roots, tubers, and bananas. Currently, Africa produces more VPCs than any other crop, including cereals and the total output of cassava alone is in fact comparable to the total output of cereals. In 2021 the total output of cassava, VPCs (including cassava) and cereals in Africa were 204, 384 and 216 million tonnes, respectively. Since 1960, the yield gap of VPCs between Africa and Asia increased by more than sixfold (FAO, 2023).

2 In particular, in the presence of asymmetric quality information, individuals are routinely forced to make decisions under partial instead of full information sets, relying heavily on perceptions and expectations (Canavari et al., 2019; Malone and Lusk, 2018; Cason and Plott, 2014). In essence, the absence of misperception implies either individuals can infer the unobservable quality from observable quality attributes accurately or there is no quality uncertainty in the sense that individuals are fully aware of true seed quality.

3 Broadly, seed quality is captured by four main attributes: genetic quality, physiological quality, seed health, and physical quality. Genetic quality refers to the traits embodied in the variety (its genotype) that are expressed when cultivated. In such cases, it is the physiological, health, and physical quality attributes—attributes that include the shape and size of the stem as well as the absence of pests and disease on or in the stem—that are more determinant of its performance as planting material, irrespective of genetics/variety type. Throughout this paper, we distinguish between “genetic quality” as described above, and “physical quality” which combines the physiological, physical and health quality attributes of stems, irrespective of genetics. If a farmer plants stems of a new variety, they may obtain gains from both its genetic quality (i.e., desirably traits) and its physical quality (especially if it is a high-quality stem). If a farmer plants recycled stems from a known source and variety (e.g., their own farm), no new information about genetic quality is obtained because propagation from the stem of the cassava plant results in a genetically identical plant (a clone).

4 Due to their inability to accurately observe and assess quality at the point of exchange, farmers then internalize the risk of deception and reduce their demand for high-quality seed.

5 TME 419 is a variety that is resistant to cassava mosaic disease (CMD) and is high-yielding when cultivated in accordance with recommended agronomic practices. TME 419 is well-known in the study area and is among the most demanded varieties among farmers in this area. The variety is also preferred for its qualities as starch, flour, *gari, fufu* (a pounded and boiled starchy provision consumed as a starchy staple), and in fresh consumption.

6 For a given variety (for a given genetic quality), the physical, physiological, and health attributes of certified seed are superior to recycled/uncertified seed (i.e., because seeds that go through a quality assurance process meet the minimum thresholds for physical, physiological and health quality attributes). Hence, the yield potential of certified TME 419 stems is higher than recycled TME419 stems. Similarly, due to its superior genetic quality, the yield potential of recycled TME419 stems is higher than recycled local variety stems.

7 At the time this experiment was conducted, the exchange rate was approximately USD 1 = NGN 385 (Nigerian Naira).

8 We opted for SPA instead of a Becker–DeGroot–Marschak (BDM) auction simply for logistical reasons associated with the bulkiness and perishability of the cassava stems. Since we cannot predict the number of potential winners and hence the amount and type of stems required in a BDM auction, it would be difficult logistically to (i) procure the required type and bundles of stems for the auction (ii) preserve and transport these stems to the location of the auction (due to bulkiness and perishability). Note that both the SPA and BDM auctions are incentive compatible, but in our case the SP mechanism overcomes the logistical problem posed by the bulkiness of cassava stems.

9 As shown in Table A1 in the appendix, the order of letter labels (T1, T2, T3) is uncorrelated with bid outcomes.

10 Own context may include, *inter alia*, their experience with cassava cultivation, available productive assets, access to factor markets, quality of their land and soil, or their own expectations.

11 The average yield values relayed to participants were as follows: 23 t/ha for certified TME 419; 14 t/ha for recycled TME 419 and 7 t/ha for local seed. Note that, while the genetic component of our comparison involves differences between two genetic lines (TME 419 vs. local varieties in our case), differences between the stems of a single genetic line (certified vs. recycled TME 419 stems in our case) comprise the physical quality component.

12 In the context of our study, we focus on cash/liquidity instead of credit constraints since seed often accounts a very small share of the total cassava production costs and hence the need or demand for credit is often low (see Karlan et al., 2014).

13 For the payment treatment intervention, our sample size is powered to detect at least a 15% increase in participants mean bid for certified improved seed. Based on data from Wossen et al. (2017) and Oparinde et al. (2016), the assumed mean and standard deviation of the expected bids were 300 and 140 NGN/bundle, respectively. Setting power = 0.8, alpha = 0.05, intra cluster correlation = 0.05, and an average size of an auction session = 11 or 7 per auction group, the required sample size for 15% MDE is: 210 individuals per arm (420 in total). While the random variation in the timing of the unconditional cash grant was introduced to address issues related to the liquidity constraint, it can also be used to explore the presence of an “experimenter demand” and/or “house money” effects. Note that neither the participants nor the enumerators were informed about the cash grant before the auction date.

14 An important aspect of our experimentally generated panel data on bid outcomes is that the time variation is minimal as the pre-and post-information rounds were separated by only a few minutes. As such, participant-level behavioural factors and unobserved heterogeneity are likely to be truly time invariant in our context.

15 Note that ψ in of Eq. (1) cannot be identified in the fixed effects specification since payment treatment status does not vary by round.

16 Note that, for each seed type the seed-specific single-difference coefficient ($\gamma_{s}+\varphi$) measures the within-stem type bid revision induced by the provision of quality information. We expect this parameter to be to be statistically insignificant when bid revision is unrelated to the quality information conveyed to participants.

17 Farmers seed quality rating based on the physical appearance of the displayed stems was identical with $p_{is1}$.

18 Our aim here is to test whether stem valuations change based on unobserved (to the farmer) seed quality, and we do not claim that information is the only variable that can affect these valuations from one round to the next. For example, the provision of quality-related information may have various behavioral effects that may influence bidding behavior. However, many of the behavioral factors are unlikely to generate systematic bid revisions in our context: behavioral mechanisms (if any) would be captured by an individual fixed effect and is unlikely to change responses to information provision. This would likely apply to both rounds. That is, we expect these variables to cause random under- or over-bidding patterns or reductions in noise between rounds, but not systematic bid revision behavior that appears as quality misperception in our data. We thank an anonymous reviewer for highlighting this detail.

19 We do not claim here that farmers can distinguish genotypes better than they can phenotypes. Rather, since the genotype of the certified and non-certified improved variety stems used in our experiment are identical (i.e., both are TME 419), the stems will inevitably look similar in appearance when compared to the local variety stems (see Figure SA2 in the appendix). This may not be the case had we varied both the variety type and certification status of the improved variety stems used in our experiment (e.g., certified TME 419 vs. recycled TMS 30572 stems or vice versa; note that there are several improved cassava varieties in Nigeria, each with relatively distinct appearances and traits). We fixed the identity of the improved variety (TME 419) to ensure that the main source of variation in farmers' valuations of certified and non-certified improved variety stems is the physical, health, and physiological quality attributes, rather than the genetic attributes. We did so in recognition of the fact that different varieties possess distinct traits, thus differing in attributes relate to their production, consumption, and processing.

20 Additional regression results are reported in Table A3 in the appendix.

21 Additional regression results, focusing on the standard deviation and coefficient variation of bid outcomes, are reported in Table A4 in the appendix.

22 For each stem type, Figure A1 in the appendix shows a histogram of the relative pdf of the pre- and post-reveal bid.

23 Note that the within-stem type distributional equality tests are based on the Wilcoxon signed rank test to account for the fact that the stem-specific bids of a given participant across the two rounds are paired. Figure A3 in the appendix shows the CDF of bid revision.

24 No good reason to expect cash grants to affect bid revision since they received the grant at the beginning (before round 1) and not at the time of bid revision (round 2).

25 According to the official certified seed production records of NASC, about 140,000 bundles of cassava stems were certified for sale in 2019. Considering the total cassava area in the country, the total cassava area planted with certified stems of improved varieties would be less than 1%.

26 Note that, due to the discrete nature of true stem quality, quality misperception, defined as the difference between the rank order of observed bid outcomes and true stem quality, is inherently non-classical in nature.

27 Distribution of pre-and post-reveal bids by cash grant treatment is reported in Figure A4 in the Appendix.

28 Table A5 in the appendix reports additional results using $(p_{is2}-p_{is1}$) as a measure of quality prediction error. Estimates based on the rank order of participants yield expectations were almost identical with the rank order of their bids.

29 Moreover, participant-level characteristics, including the level of education, were unrelated to the bid revision pattern observed in our data.

30 Note that, post-quality information, we expect all participants to bid higher or equal amount for certified improved stems relative to recycled improved stems (i.e., at the same price, certified TME 419 is preferred to the non-certified TME 419).

31 We refer these group as “defiers.”

32 While certification offers a solution to the information problem, it can also break down in the presence of poor/weak quality control-where regulators have limited capacity to implement, monitor, or enforce quality standards effectively. Without credible quality signalling mechanisms, our experimental finding suggest that the seed system should be viewed almost exclusively as a distribution channel for stems of existing (and new) improved varieties at any quality that can provide farmers with a wider choice of agronomic, stress-resistance, nutritional, and processing traits. More specifically, with our experimental finding that improved non-certified stems (medium quality) are correctly valued without additional information on stem quality, informal stem markets could be leveraged on to increase the overall supply of stems of improved varieties at prices that are closer to the willingness to pay of farmers, thereby reducing the share of farmers relying on local varieties and ultimately improving adoption and cassava yields. Considering the low adoption and slow varietal turnover rate of improved varieties in Nigeria, efforts to increase access to improved varieties (medium-quality) through strategic investment and support to seed entrepreneurs (e.g., training on seed production, internal inspection practices, business planning and marketing, preferential access to early generation seed or access to mass propagation technologies etc.) could play an important role in accelerating both adoption and varietal turnover (i.e., switching from local to improved varieties) in the country. That said, at upper levels in the seed system—where breeder and foundation seed are produced and distributed—the quality of planting material needs to be managed carefully to avoid potentially catastrophic introduction of new pests and diseases.
