# Supplementary material for: Estimating seed demand in the presence of market frictions: Evidence from an auction experiment in Nigeria
Source: J Dev Econ. 2024 Mar;167:103242. doi: 10.1016/j.jdeveco.2023.103242 (PMC10831485; doi:10.1016/j.jdeveco.2023.103242)
Supplement: Multimedia component 1 [file mmc1.docx]

## [Additional Information](https://www.dropbox.com/s/nim0efz5jag0ac6/Additional_Material.pdf?dl=0)

**Figure SA1: An example of soap bars and cassava stems used in the practice and seed auctions**


**Figure SA2: An example of the actual seed auctions without and with quality information provision**
